# The Relationship of DNA Methylation with Age, Gender and Genotype in Twins and Healthy Controls

**DOI:** 10.1371/journal.pone.0006767

**Published:** 2009-08-26

**Authors:** Marco P. Boks, Eske M. Derks, Daniel J. Weisenberger, Erik Strengman, Esther Janson, Iris E. Sommer, René S. Kahn, Roel A. Ophoff

**Affiliations:** 1 Rudolf Magnus Institute of Neuroscience, Department of Psychiatry, University Medical Center Utrecht, Utrecht, The Netherlands; 2 Julius Centre for Health Sciences and Primary Care, University Medical Center Utrecht, Utrecht, The Netherlands; 3 USC Epigenome Center and USC Norris Comprehensive Cancer Center Keck School of Medicine, University of Southern California Los Angeles, Los Angeles, California, United States of America; 4 Department of Medical Genetics, University Medical Center Utrecht, Utrecht, The Netherlands; 5 UCLA Center for Neurobehavioral Genetics, Semel Institute for Neuroscience and Human Behavior, Los Angeles, California, United States of America; City of Hope Medical Center, United States of America

## Abstract

Cytosine-5 methylation within CpG dinucleotides is a potentially important mechanism of epigenetic influence on human traits and disease. In addition to influences of age and gender, genetic control of DNA methylation levels has recently been described. We used whole blood genomic DNA in a twin set (23 MZ twin-pairs and 23 DZ twin-pairs, N = 92) as well as healthy controls (N = 96) to investigate heritability and relationship with age and gender of selected DNA methylation profiles using readily commercially available GoldenGate bead array technology. Despite the inability to detect meaningful methylation differences in the majority of CpG loci due to tissue type and locus selection issues, we found replicable significant associations of DNA methylation with age and gender. We identified associations of genetically heritable single nucleotide polymorphisms with large differences in DNA methylation levels near the polymorphism (*cis effects)* as well as associations with much smaller differences in DNA methylation levels elsewhere in the human genome (*trans effects)*. Our results demonstrate the feasibility of array-based approaches in studies of DNA methylation and highlight the vast differences between individual loci. The identification of CpG loci of which DNA methylation levels are under genetic control or are related to age or gender will facilitate further studies into the role of DNA methylation and disease.

## Introduction

Epigenetic factors have recently been found to play an important role in developmental plasticity [1]–[3]. The molecular mechanisms that underlie the epigenetic modifications of phenotype are increasingly well understood. Coordinated changes in the methylation of cytosine-5 at CpG dinucleotides in the promoter regions of specific genes, changes in chromatin structure through histone acetylation and methylation and post-transcriptional control by microRNAs [4] have been shown to alter gene transcription levels. Specifically, methylation of CpG-rich promoter areas (CpG islands) has been identified as an important mechanism in the dynamic regulation of gene transcription of mammalian genes [5], [6]. DNA methylation is established early in embryogenesis [7] and is the prime mechanism of X-chromosomal inactivation as well as of imprinting effects [8]. Differential DNA methylation can be age-related [9], [10], and is involved in a number of disorders including human cancers [11]. As DNA methylation plays an important role in the mechanism of epigenetic memory [12] it is of primary interest for the study of epigenetic influences on human traits and diseases.

Studies involving monozygotic and dizygotic twins have been used to investigate the influence of DNA methylation on phenotypic variation. A study using monozygotic twins discordant for caudal duplication anomaly revealed significant differences in DNA methylation patterns of the disease associated gene *AXIN1* (NC_000016) in peripheral blood [13]. In monozygotic twins discordant for low birth weight, differential DNA methylation was observed in the *COMT* gene region (NC_000022), suggesting that DNA methylation may provide a mechanism through which birth weight affects the risk for schizophrenia [14]. Several studies have since tried to find differences in DNA methylation in twins discordant for schizophrenia with mixed results [15]–[18]. Analysis of the heritability of DNA methylation in the human *IGF2/H19* locus (NC_000011) in twins revealed substantial heritability and *cis* genetic associations with single nucleotide polymorphisms (SNPs) [19]. The first systematic evidence for familial clustering of DNA methylation presented longitudinal DNA methylation changes as well as genetic influences [20]. Further evidence that DNA methylation patterns are heritable came from a recent twin study using white blood cells and buccal tissue although this study suggested that the molecular mechanisms of heritability may not be restricted to DNA sequence differences [21].

Based on these recent findings a picture is emerging in which DNA methylation levels at specific loci are to some degree under genetic control [19], [21]–[23] and subject to changes over time. Previous studies observed that patterns of epigenetic modification diverge in twins as they become older and suggest that this could be due to small defects in transmitting epigenetic information through successive cell divisions, or maintaining it in differentiated cells [24], [25]. This process, termed “epigenetic drift”, is associated with the aging process. In addition to the reported associations of DNA methylation levels with age and genotype, there is evidence that methylation levels are related to gender. There are several studies that found higher global DNA methylation levels in males [22]. Studies on gender-associated differences in DNA methylation at specific loci have yielded contrasting results [26].

To investigate the heritability of DNA methylation as well as the effects of age and gender, we performed a pilot study of DNA methylation in healthy twin and control subjects using the Illumina GoldenGate Methylation assay. In this assay 1,505 CpG sites from more than 800 genes in the human genome are interrogated simultaneously. The aim of the study was two-fold. First, we were interested to see whether it was feasible to successfully measure DNA methylation levels in blood using commercially available arrays. Second, we determined whether previously reported effects of age, gender and genotype on DNA methylation levels were restricted to specific loci and whether the effects sizes were large enough to be detected.

## Results

### DNA Methylation analysis

We generated DNA methylation data of MZ twins (N = 43), DZ twins (N = 43) and 96 healthy singletons using peripheral blood DNA samples and the GoldenGate Methylation assay. First, we used BeadStudio software to obtain intensity measures (the mean cy3+cy5 value) for each sample. Using a threshold intensity value of 4,000, we eliminated eight subjects from five pairs (including replicate pairs) in the twin set from further analysis. We next excluded eight probes that had a detection p-value>0.05 in more than 5 % of the hybridizations. The failure rate in the remaining 1,497 probes was <0.01 %. We repeated the above steps for the 96 healthy control subjects included in the second set. In the latter series, 11 of the 1,505 DNA methylation probes and one subject failed quality control and were removed from further analysis.

We performed two-dimensional unsupervised hierarchical clustering of autosomal loci in the filtered data set (Heatmap and histogram shown in Supplementary Figures S1 and S2). These analyses showed a bimodal distribution of DNA methylation [27]. A large proportion of the probes showed no or very little variation in DNA methylation levels and were either fully methylated or unmethylated. Since Illumina arrays can only reliably estimate DNA methylation differences greater or equal to 17 percent only probes with a sufficient range were included for further analysis (See methods). From the 411 probes with normal distribution in both sets as well as in the combined set, 131 failed the range criterion leaving 280 high quality probes in 250 genes for further study of heritability, age and gender effects.

### Age-related DNA methylation

We investigated the association between DNA methylation levels of the 280 high-quality probes with age. We observed a strong correlation between the two experiments with an overall Spearman correlation for t-values of 0.77 (95% CI: 0.71–0.81). The combined analyses identified a total of 58 probes that were significantly related to age (21.5 percent). Six loci also showed a significant correlation with age in both samples series independently (Table 1). The genes represented by these probes were activin A receptor type I (*ACVR1*) (NM_001105.2), interleukin 6 (*IL6*) (NM_000600.1), caspase recruitment domain-containing protein 15 (*CARD15*) (NM_022162.1), platelet-derived growth factor receptor alpha (*PDGFRA*) (NM_006206.3), nuclear factor kappa-B subunit 1 (*NFKB1*) (NM_003998.2), and ETS-domain protein (*ELK*) (NM_005229.2). The associations with age of all 251 DNA methylation probes are listed in Supplementary Table S1.

**Table 1:** DNA methylation probes with significant effects of age and gender (listed are genes for which significance was detected in both series independently).

	Range		Twins				Singletons							
Probe	min	max	B.x	Chi2.x	p.x	p.fdr.x	B.y	Chi2.y	p.y	p.fdr.y	Symbol	GenBank	Chr	Locus
**Age**														
ACVR1_E328_R	0.78	0.99	−0.403	11.361	7.50E-04	3.88E-02	−0.648	55.757	8.20E-14	2.30E-11	*ACVR1*	NM_001105.2	2	158402708
IL6_E168_F	0.00	0.32	0.476	18.525	1.68E-05	3.86E-03	0.443	37.288	1.02E-09	9.51E-08	*IL6*	NM_000600.1	7	22733513
CARD15_P302_R	0.17	0.61	−0.390	11.309	7.71E-04	3.88E-02	−0.440	33.265	8.04E-09	5.63E-07	*CARD15*	NM_022162.1	16	49288249
PDGFRA_E125_F	0.25	0.98	0.471	17.580	2.75E-05	3.86E-03	0.412	30.935	2.67E-08	1.29E-06	*PDGFRA*	NM_006206.3	4	54790329
NFKB1_P496_F	0.08	0.50	−0.422	15.125	1.01E-04	9.39E-03	−0.361	30.863	2.77E-08	1.29E-06	*NFKB1*	NM_003998.2	4	103641022
ELK3_P514_F	0.01	0.28	0.385	11.172	8.30E-04	3.88E-02	0.380	28.501	9.37E-08	3.75E-06	*ELK3*	NM_005230.2	12	95111824
**Gender**														
TDGF1_P428_R	0.22	0.64	0.791	10.777	1.03E-03	2.88E-02	1.050	41.315	1.30E-10	1.81E-08	*TDGF1*	NM_003212.1	3	46593789
EVI2A_E420_F	0.27	0.97	0.816	12.815	3.44E-04	1.60E-02	0.796	30.494	3.35E-08	2.34E-06	*EVI2A*	NM_001003927.1	17	26672423
NDN_E131_R	0.54	0.87	1.028	23.063	1.57E-06	4.39E-04	0.487	26.130	3.19E-07	1.79E-05	*NDN*	NM_002487.2	15	21483412
PLAGL1_E68_R	0.63	0.91	0.883	17.177	3.41E-05	4.77E-03	0.628	24.507	7.40E-07	3.46E-05	*PLAGL1*	NM_002656.2	6	144371178
DNMT2_P199_F	0.75	0.96	0.858	13.209	2.79E-04	1.56E-02	0.686	23.858	1.04E-06	4.15E-05	*DNMT2*	NM_004412.3	10	17283886
ERN1_P809_R	0.18	0.58	−0.868	13.505	2.38E-04	1.56E-02	−0.468	22.692	1.90E-06	6.66E-05	*ERN1*	NM_001433.2	17	59562017
PTHR1_P258_F	0.63	0.86	0.720	9.892	1.66E-03	3.87E-02	0.611	17.822	2.43E-05	6.17E-04	*PTHR1*	NM_000316.2	3	46893982
XPC_P226_R	0.75	0.99	0.904	14.454	1.44E-04	1.34E-02	0.553	20.202	6.97E-06	1.95E-04	*XPC*	NM_004628.3	3	14195369
PYCARD_P150_F	0.18	0.48	0.756	11.670	6.35E-04	2.22E-02	0.263	10.500	1.19E-03	1.19E-02	*PYCARD*	NM_145182.1	16	31121902
SGCE_P250_R	0.33	0.97	0.803	11.878	5.68E-04	2.22E-02	0.279	9.005	2.69E-03	1.98E-02	*SGCE*	NM_003919.1	7	94123664

### Gender-related DNA methylation

We next investigated the relationship of DNA methylation levels with gender. As expected, non-parametric analysis with Mann-Whitney U test showed that all X-chromosomal probes on the bead array were significantly more methylated in females. However, all but two X-linked probes failed the stringent normality test. For this reason we restricted the analysis of gender effects to the autosomal loci only. A total of 56 of the autosomal probes (20%) showed a significant effect of gender in the combined samples (see Supplementary Table S2 for complete list). The DNA methylation status at ten loci was also significantly associated with gender in the twin and singleton samples independently (Table 1). Although highly significant, the mean difference in DNA methylation level even at the most significant locus (TDGF1_P248_R, NM_003212.1) in the combined group was modest (0.75, sd 0.04 in females compared to 0.79, sd 0.04 in males. t = 4.5734, df = 169.39, p = 9.23e–06). Similar to the age-specific findings, we observed fairly good overall correlation of the gender effects on DNA methylation levels in the two sample sets at 0.69 (95% CI: 0.62–0.74).

### Heritability

Third, the twin sample was used to obtain heritability estimates of the observed high-quality DNA methylation profiles. In total, 96 of the 431 CpG sites (23%) yielded a significant heritability (Table 2). The most significant heritable DNA methylation levels included sites in AXL receptor tyrosine kinase isoform (*AXL*) (NM_001699.3), glutathione S-transferase M1 isoform 2 (*GSTM1*) (NM_000561.2), T-cell lymphoma invasion and metastasis 1 (*TIAM1*) (NM_003253.2) and TEK tyrosine kinase (*TEK*) (NM_000459.2) among others (See supplementary table S3 for full heritability result).

**Table 2:** Heritability of probes (top 25 only).

Probe	Her	Chi2	p-value	Symbol	GenBank	Chr	Locus
AXL_P223_R	0.94	54.390	1.64E-13	*AXL*	NM_021913.2	19	46416440
GSTM1_P363_F	0.90	36.833	1.29E-09	*GSTM1*	NM_146421.1	1	110031602
TIAM1_P188_R	0.76	23.136	1.51E-06	*TIAM1*	NM_003253.1	21	31853349
CHGA_P243_F	0.87	20.119	7.28E-06	*CHGA*	NM_001275.2	14	92459002
TEK_P479_R	0.72	19.197	1.18E-05	*TEK*	NM_000459.1	9	27098962
HPN_P823_F	0.76	19.065	1.26E-05	*HPN*	NM_182983.1	19	40222427
CREBBP_P712_R	0.73	17.853	2.39E-05	*CREBBP*	NM_004380.1	16	3871424
JAK3_P1075_R	0.67	16.326	5.33E-05	*JAK3*	NM_000215.2	19	17820875
NPR2_P618_F	0.64	16.126	5.93E-05	*NPR2*	NM_003995.3	9	35781788
CCL3_E53_R	0.77	15.769	7.16E-05	*CCL3*	NM_002983.1	17	31441547
NPR2_P1093_F	0.57	15.696	7.44E-05	*NPR2*	NM_003995.3	9	35781313
HRASLS_P353_R	0.66	15.472	8.37E-05	*HRASLS*	NM_020386.2	3	194441259
MET_E333_F	0.64	15.122	1.01E-04	*MET*	NM_000245.2	7	116100028
HOXA5_P1324_F	0.68	15.054	1.04E-04	*HOXA5*	NM_019102.2	7	27151136
TSP50_P137_F	0.73	14.607	1.32E-04	*TSP50*	NM_013270.2	3	46734505
FZD9_E458_F	0.66	14.545	1.37E-04	*FZD9*	NM_003508.2	7	72486503
CRK_P721_F	0.60	14.229	1.62E-04	*CRK*	NM_005206.3	17	1307015
HHIP_P578_R	0.63	13.141	2.89E-04	*HHIP*	NM_022475.1	4	145786045
IL16_P226_F	0.59	12.836	3.40E-04	*IL16*	NM_004513.3	15	79262029
ACVR1_E328_R	0.64	12.729	3.60E-04	*ACVR1*	NM_001105.2	2	158402708
DNAJC15_E26_R	0.67	12.536	3.99E-04	*DNAJC15*	NM_013238.2	13	42495388
ABCC2_E16_R	0.58	12.367	4.37E-04	*ABCC2*	NM_000392.1	10	101532577
AXIN1_P995_R	0.61	12.179	4.83E-04	*AXIN1*	NM_003502.2	16	343460
DDR1_P332_R	0.67	12.085	5.08E-04	*DDR1*	NM_001954.3	6	30959508
LIG3_P622_R	0.57	11.919	5.56E-04	*LIG3*	NM_013975.2	17	30331029

### Linkage Disequilibrium mapping

**Figure 1:**
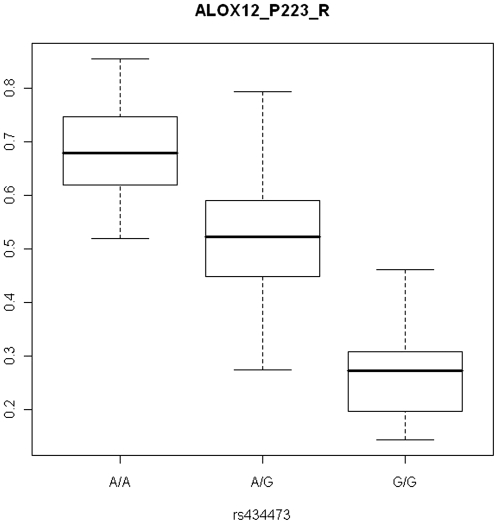
Sample plot of DNA methylation levels at probe ALOX12_P223_R by genotype of SNP rs434473.

Based on the high heritability estimate of DNA methylation profiles of multiple CpG sites, we performed association mapping using DNA methylation levels as quantitative traits. For 91 subjects of the second set, sample genotype data was available (Illumina 550K BeadChip), with a total of 437,968 SNPs genome-wide after quality control filtering. Genomic inflation factor as a measure of population stratification ranged from 0.96 to 1.05, the mean chi-square statistic ranged between 0.98 and 1.03 indicating an absence of population stratification. We observed significant associations between DNA methylation at 11 different CpG loci representing 35 *cis* and 5 *trans* effects after stringent multiple testing correction and confirmation by permutation testing (Supplementary Table S4 shows all 106 methylation probes that showed significant associations after FDR correction for multiple testing, uncorrected for the 512 phenotypes tested). It is noticeable that all significant *trans* effects are observed with common variants located within genes, and not positioned in regions with known micro RNA's (http://microrna.sanger.ac.uk/). We here present genetic associations of three DNA methylation loci with nine SNPs that constituted a mean difference in DNA methylation level larger or equal than 0.17 to rule out the possibility of technical artifact and survived corrections for multiple testing both for the number of SNPs as well as the 512 phenotypes employed (Table 3). An example plot of DNA methylation differences of *ALOX12* (NM_000697.1), the gene locus with the most significant association of DNA methylation with genotype, is shown in Figure 1. The heritability of these CpG loci as estimated in the twin data by means of non-parametric analysis (polychoric correlations) was significant for ALOX12_E85_R (heritability: 0.48, 95% CI: 0.24–0.61) and KRAS_E82_F (heritability: 0.9 95% CI: 0.79–1), but non-significant for ALOX12_P223_R (heritability: 0.03, 95% CI: 0–0.3) or MET_E333_F (heritability: 0.07 95% CI:0–0.42). Associations of DNA methylation level of two X- chromosomal CpG loci (*GRPR* (Xp22.2, NM_005314.2), and *PCTK1* (Xp11.3, NM_033018.2)) with the SNPs rs12743401, rs3881953, rs12734338 rendered non-significant after including gender as a covariate. The association signals are most likely from a homolog area on the Y chromosome as the association of these SNPs with male gender (p<1E-8) confirms.

**Table 3:** Genetic associations with DNA methylation levels.

Probe	CHR probe	SNP	CHR	Base	MAF	AA Mean (sd)	AB Mean (sd)	BB Mean (sd)	BETA	SE	R2	T	p	FDR
ALOX12_E85_R	17	rs434473	17	A/G	0.49	0.649 (0.133)	0.493 (0.142)	0.303 (0.151)	−0.173	0.023	0.395	−7.629	2.49E-11	6.37E-03
ALOX12_E85_R	17	rs1126667	17	A/G	0.50	0.315 (0.156)	0.492 (0.143)	0.649 (0.133)	−0.167	0.023	0.380	−7.383	7.86E-11	2.01E-02
ALOX12_P223_R	17	rs434473	17	A/G	0.49	0.686 (0.093)	0.523 (0.120)	0.277 (0.112)	−0.204	0.018	0.583	−11.160	1.33E-18	3.40E-10
ALOX12_P223_R	17	rs1126667	17	A/G	0.50	0.291 (0.127)	0.522 (0.121)	0.686 (0.093)	−0.198	0.019	0.560	−10.650	1.50E-17	3.82E-09
ALOX12_P223_R	17	rs2292350	17	A/G	0.38	0.693 (0.081)	0.558 (0.112)	0.372 (0.180)	0.168	0.022	0.391	7.554	3.53E-11	9.03E-03
MET_E333_F	7	rs38840	7	C/T	0.20	0.835 (0.004)	0.701 (0.062)	0.575 (0.084)	0.127	0.015	0.452	8.567	2.95E-13	7.54E-05
MET_E333_F	7	rs1002399	7	C/T	0.21	0.835 (0.004)	0.694 (0.067)	0.575 (0.086)	0.121	0.015	0.417	7.976	4.87E-12	1.25E-03
KRAS_E82_F	12	rs1566753	6/12	G/T	0.37	0.606 (0.067)	0.518 (0.065)	0.433 (0.074)	−0.087	0.010	0.461	−8.719	1.44E-13	3.67E-05
KRAS_E82_F	12	rs4516942	6/12	A/G	0.37	0.606 (0.067)	0.518 (0.065)	0.433 (0.074)	−0.087	0.010	0.461	−8.719	1.44E-13	3.67E-05

### Sequencing

To investigate whether sequence variation at the probe target regions may affect hybridization and cause spurious association findings, we performed a targeted sequencing effort of 15 gene regions. All subjects of the second set were included for sequencing *EVI2A* (NM_001003927.1), which showed high heritability and strong associations with DNA methylation. We used a subset of subjects representing the upper and lower quartile of DNA methylation values and homozygosity of the associated alleles for four additional gene regions (ALOX12_P223_R, TDG_E129_F, CTSD_P726_F, TMPRSS4_P552_F). We observed one novel sequence variation within the EVI2A_E420_F probe region in one subject. The remaining six loci did not show any genetic variation. (Detailed results of sequence data are available upon request.)

## Discussion

This study surveys inter-individual DNA methylation in relation to genotype, age and gender effects simultaneously. Using genomic DNA extracted from peripheral blood, we analyzed DNA methylation patterns of a large number of CpG sites from more than 800 genes spread throughout the genome in 23 monozygotic and 23 dyzygotic healthy twin-pairs and 96 healthy singletons. We demonstrate that the application of readily available array based technology can provide meaningful results that open the opportunity of incorporating epigenetic information in genetic studies.

Despite the fact that in the majority (86 percent) of CpG sites interrogated by this panel, no substantial DNA methylation variation was present due to tissue type and CpG selection, we did observe replicable, independent, significant effects of age and gender on DNA methylation status for some of the remaining sites. Furthermore, for a number of DNA methylation loci in the twin data, we observed high heritability values for their DNA methylation levels. Genome-wide mapping using DNA methylation as a quantitative trait in 91 healthy singletons with available SNP data yielded highly significant associations. Particularly some of the *cis* effects constituted large effect sizes that are likely to be biologically relevant and survived strict correction for multiple testing. Other associations, although statistically significant, constituted smaller effect sizes and included *trans* effects of genetic variation on DNA methylation levels as well. Additional sequence analysis did not identify DNA variation within the targeted CpG probe regions that could have lead to spurious association findings [28]. Our results in the twin data as well as in the singletons demonstrate the role of genetic variation regulating epigenetic signals.

The strongest effects of genetic regulation act locally (*cis*), within regions that are in linkage disequilibrium with the CpG sites assayed on the array. However, we also observed a number of significant genetic associations with DNA methylation status elsewhere in the genome on other chromosomes (i.e. *trans* effects) albeit with small effect sizes and with unknown biological relevance. Given the fact that *trans* effects were already observed in a relatively small sample size of 91 subjects with limited power in a small subset of CpG methylation sites, it is likely that larger studies will identify more *trans* effects and uncover new genetic regulation of epigenetic profiles. Further non-parametric analysis of association signals may implicate additional genetic control of methylation levels. It is noticeable that all significant *trans* effects are observed with common variants located within genes instead of gene deserts, suggesting biologically relevant interactions. However caution is warranted as some *trans* effects were identified as artifacts. For instance the genetic associations in *trans* from the X-chromsomal CpG sites were due to homologs of the associated SNPs on the Y-chromosome and the SNPs (rs1566753, rs4516942) listed at chromosome 6 but have homologs at chromosome 12 and therefore most likely constitute *cis* effects rather *trans* effects on methylation levels of the *KRAS* locus (NM_033360.2) on chromosome 12. However, another *trans* effect such as from rs3134269 in the Solute Carrier Family 25, member 32 gene (SLC25A32; NC_000008.10) (involved in folate metabolism) that is associated with DNA methylation levels at *TSC2* (tuberous sclerosis 2, NM_001077183.1) constitute a difference of 0.24 percent, well above detection sensitivity (Supplementary Table S5). We are not aware of any studies reporting a direct role of this gene in DNA methylation, but our results suggest that further studies into the molecular mechanisms of these trans effects are warranted.

Recent studies have begun to reveal the complex underlying control mechanisms of DNA methylation but studies of the effect of sequence variant on DNA methylation levels have yielded contrasting results [21], [29], [30]. Our data provides evidence for *cis*-acting DNA sequences that exert local epigenetic control possibly extending over a cluster of nearby genes and suggest the presence of *trans*-acting factors not limited to DNA methyltransferases [31]. The SNPs associated with methylation levels in our study were not positioned in genomic regions with microRNAs and we therefore are unable to corroborate recent studies suggesting that microRNAs are associated with DNA methylation levels [32], [33].

In addition to genetic control of DNA methylation levels, we found independent associations of DNA methylation with age and gender. Gender effects for methylation loci on the X chromosome are largely due to the X-inactivation dosage compensation mechanism in females [34]. However, we observed that several of variable autosomal CpG loci on the array are differentially methylated in females as well. We are not the first to report gender specific DNA methylation differences suggesting that autosomal gender dependent DNA methylation is a recurrent phenomenon. Although the absolute differences in methylation level between male and female participants was low, nevertheless they constitute effect sizes similar to previous findings from other groups (Cohen's d = 1) [26]. Our study is also in agreement with previous reports that DNA methylation is age related although this may be restricted to mitotic cells [35] and specific loci [19]. It is noteworthy that about half of the probes that showed significant association to age and gender are non-CpG island probes. The ratio of CpG island in the samples that showed approximate normal distribution and sufficient range (173 of 280) was significant lower than in the original set (1044 of 1505) (Chi-square test: Χ^2^ = 5.9, df = 1, p = 0.015). These results support recent reports that changes in DNA methylation do not predominantly occur in promoter regions or CpG islands [36].

The DNA methylation loci with high heritability or with significant associations with age or gender identified in our study present some genes that have previously been implicated in disease. For example, the *PTHR1* (parathyroid hormone receptor 1, NM_000316.2) gene that was found differentially methylated in females is associated with bone mineral density that shows substantial difference between males and females. Sequence variation of the *IL6* gene (NM_000600.1) is related to metabolic syndrome in older males. Finally the *AXIN1* locus (NM_003502.2) of which DNA methylation levels in peripheral blood has previously been associated with caudal duplication anomaly showed high heritability in our sample (heritability: 0.61, Χ^2^ = 12.18, p<0.001). Further studies into the phenotypic consequences of such methylation differences seem warranted.

We realize that these findings are based on a relatively small sample size with limited statistical power. In addition, the Illumina GoldenGate Cancer Panel probes represent only a minute fraction (less than 0.005 percent) of all CpGs in the human genome. On estimate there are 32 million CpG dinucleotides constituting approximately 1 one percent of the genome [37], [38]. The selection of CpG probes predominantly in promoter regions of cancer related genes may not be representative for the vast number of DNA methylation sites throughout the genome and are known to be commonly unmethylated in blood [6], [27]. A recent study of the human B cell methylome suggest that DNA methylation at different regions in the genome (whether in the promoter, intragenic or near the 3′ end) exert different effects on gene transcription [39]. Selection of probes with substantial range and proximal normal distribution as employed in this study focuses on the strongest and biologically most relevant signals. Further non-parametric analysis may tease out additional relevant signals but is beyond the scope of this paper. Technological array-based advances will enable us to study a much larger number of targeted CpGs, while high-throughput, next-generation sequencing studies will provide genome-wide DNA methylation information. Such new developments may also overcome the current limitations in detection sensitivity of the Illumina GoldenGate panel applied in this study. The arrays can detect DNA methylation levels as low as 2.5 percent, but are generally more accurate detecting higher levels of methylation (above 10 percent). Bibikova and colleagues suggested that a beta value difference of ≥0.17 is needed in analyzing GoldenGate methylation data [40]. Whereas the sensitivity to detect differences greater than this difference is excellent, caution is warranted for smaller differences. The mean differences between the genotypes presented in Table 4 are all above this detection threshold as are the ranges in DNA methylation levels that were significantly associated with age or gender. However the mean differences between the male and female groups were smaller than 17 percent and should therefore be interpreted with caution.

**Table 4:** Sample description.

	Twins		Singletons	All
	Monozygotic	Dizygotic		
**N**	46 (23 pairs)	46 (23 pairs)	96	188
**Mean Age (SD; range)**	39.5 (11.1; 20.8–57.8)	39.6 (10.0; 18.8–53.9)	34.2 (16.0; 19.6–66.2)	36.8 (13.3; 18.8–66.2)
**Male (%)**	20 (43%)	18 (39%)	48 (50%)	86 (46%)

A remaining challenge will be the study of tissue-specificity of epigenetic control. We do not know what the meaning of differential DNA methylation in blood is for other tissues. Although several studies suggest an important role of DNA methylation in the specialization of tissues [41] other studies suggest that the role of DNA methylation in tissue-specific gene expression is limited to tissue-specific differentially methylated regions (T-DMRs) [42], [43]. Our data are in agreement with those of Kaminsky *et al* who reported that the intraclass correlation between tissue type was significantly higher compared to intra-individual differences [21]. Nevertheless, further studies that include several tissue types preferably from single subjects are necessary [25]. In absence of information on the exact types of lymphocytes that were included in our blood samples this remains a potential source of bias in this study too.

DNA methylation information can potentially be applied to the study of environmental influences, genetic mapping studies and the development of biomarkers. Further studies may unveil novel mechanisms by which the environment exerts influence on the susceptibility to diseases and may also be important for genetic linkage and association studies. It is possible that DNA methylation of disease-associated alleles may affect the relative risk of carriers to develop the trait, cause phenotypic heterogeneity or may explain some of the phenotypic differences in monozygous twins [44]. For this reason, incorporating DNA methylation data with association and linkage analyses may provide new insights for correlating genetic markers and disease, and thus increase the power of linkage and association studies. This pilot provides a first glimpse of the possibilities and hurdles of large-scale DNA methylation profiling.

## Methods

### Ethics statement

Written informed consent from all participants was obtained. This study was approved by the Medical Ethical Committee of the UMC Utrecht, The Netherlands.

### Subjects

The twin sample included 23 healthy monochorionic or dichorionic monozygotic twin pairs (46 subjects) and 23 healthy dizygotic twin pairs (an additional 46 subjects) recruited via advertisements in local newspapers and media. Inclusion criteria included good general health and at least three Dutch grandparents. Zygosity of twin pairs was investigated previously by the Illumina DNA Test Panel consisting of 360 validated single nucleotide polymorphisms (SNPs). General Health was established using a general medical checklist; all subjects were free of medication. An independent second sample set consisted of 96 healthy controls, matched for age and gender to the original twin series. For this second sample set, genome-wide SNP data (Illumina 550K Infinium array) was available. The second sample was used to validate the age and gender effects observed in the twin sample and to perform genetic mapping using DNA methylation as quantitative traits. The mean age and gender information for both sample sets are listed in Table 4.

### Laboratory analysis

Genomic DNA extracted from peripheral blood was used for this study. Experiments of the twin data and singletons were performed independently. Genomic DNA samples (0.5 µg) from both sets were bisulfite converted using the EZ-96 DNA methylation kit (ZYMO Research, Orange, CA, USA) according to the manufacturer's protocol in two batches, first the twins and secondly the singletons. The twins and co-twins were randomly distributed across plates. DNA methylation profiles were obtained using the commercially available Illumina GoldenGate DNA methylation platform (DNA Methylation Cancer Panel I) as recommended by the manufacturer . With this assay, the DNA methylation levels of up to 1,505 CpG sites covering 807 gene regions are interrogated concurrently. The probe IDs are listed in Supplemental Table S5 and online by the manufacturer (www.illumina.com/pages.ilmnID193).

### Statistical Analysis

We used Illumina BeadStudio software for normalization and initial quality control. BeadStudio uses a background normalization that subtracts a background value derived by averaging the signals of built-in negative control bead types. The DNA Methylation status for each probe is presented as a Beta value, the ratio of signal from the methylated probe relative to the sum of both methylated and unmethylated probes. Beta values can range from 0 (unmethylated) to 1 (fully methylated). We tested for approximate normal distribution by means of a Lilliefors test for normality at the 0.1 level after FDR correction for multiple testing. Non-normal distributed probes were excluded from subsequent analysis. To ensure biological relevance of the methylation differences, we also excluded those probes that had a range smaller than 0.17 in the methylation levels. For further analysis, we used Z-scores of DNA methylation levels and age in a variance component analysis. Analyses of age and gender effects were conducted for the twins and singletons, both separately and jointly. The effects of age and gender on the level of methylation were tested in Mx [45]. The covariance of the methylation levels in MZ and DZ twin-pairs was estimated to take into account the statistical dependency of the data. Two parameters were used to model the variances and covariances in MZ and DZ twins, representing an additive genetic factor (A), and a unique environmental factor (E). Variances in both MZ and DZ twins are explained by additive genetic variation and unique environmental variation, and are therefore estimated as A+E. The covariance in MZ twins is expected to equal A, as MZ twins share their genetic material. The covariance in DZ twins is expected to equal 0.5*A, as DZ twins share 50% of their genetic material. The heritability was estimated as the additive genetic variance (A) divided by the total amount of variance (A+E). Heritability Chi-square statistic and probability were calculated for all the probes that showed approximate normal distribution and sufficient range in the twins set. We used Mann-Whitney non-parametric testing to test for differences in DNA methylation of X-chromosomal probes between males and females, chi-square test for testing the proportion of CpG islands before and after filtering. Heritability and standard error non-normal distributed methylation levels were estimated based on an ACE model using Falconer's formulae [46]. Under the ACE model the correlation between the DZ twins is expected to be equal or higher than half of the correlation between the MZ twins.

Normalization steps were conducted in BeadStudio, variance component analysis was conducted using Mx [42] all other analyses were done using the R package for statistical computing [47].

### Association analysis

We generated SNP genotype data for 91 samples in the singletons using the Illumina HumanHapmapv1.1 550K platform at UCLA. Associations with the 512 DNA methylation levels that were normally distributed in this sample as quantitative phenotypes, were calculated using PLINK [48]. Provided the small sample size, we included only SNPs with minor allele frequency (MAF) at ≥0.1 and a genotyping success rate of >90%. Markers deviating from expected Hardy-Weinberg equilibrium at p<0.001 using an exact test [49] were also excluded. Population stratification was assessed using genomic inflation factor (based on median chi-squared) and the mean chi-square statistic. We applied FDR correction at the 0.05 level [50] for multiple testing by the number of SNPs and the number of phenotypes tested. Adaptive permutation testing (n = 10^7^) was performed for the associated SNPs to confirm our findings. These analyses were carried out on the Genetic Cluster Computer (http://www.geneticcluster.org). Association signals that were obtained from X-chromosomal loci were re-calculated using gender as covariate in a linear regression model.

### Sequencing

To investigate whether DNA sequence variation at probe regions affected the hybridization signals, we examined several candidate loci by standard sequencing. Selection of CpG sites for sequencing was based on the presence of highly significant association findings. All subjects from the second set were sequenced for PCR amplicons for the EVI2A_E420 locus; eight selected subjects were used to screen for sequence variation in the probe regions of ALOX12_P223_R, TDG_E129_F, CTSD_P726_F, TMPRSS4_P552_F. The latter subjects were selected by two criteria: DNA methylation levels in the upper or lower quintile, and homozygosity for the associated SNPs. The probes were selected from a range of strength of association signals based on the availability of suitable primers. Primer sequences are available upon request.

## Supporting Information

Table S1 Full age results
(0.12 MB XLS)

Table S2 Full gender results
(0.13 MB DOC)

Table S3 Full heritability result
(0.10 MB DOC)

Table S4 Full mapping results
(0.06 MB XLS)

Table S5 Probe IDs
(1.09 MB XLS)

Figure S1 Heatmap of twin sample
(1.28 MB TIF)

Figure S2 Heatmap of singleton sample
(1.28 MB TIF)
