# Supplementary figures and images for: The Relationship of DNA Methylation with Age, Gender and Genotype in Twins and Healthy Controls
Source: PLoS One. 2009 Aug 26;4(8):e6767. doi: 10.1371/journal.pone.0006767 (PMC2747671; doi:10.1371/journal.pone.0006767)

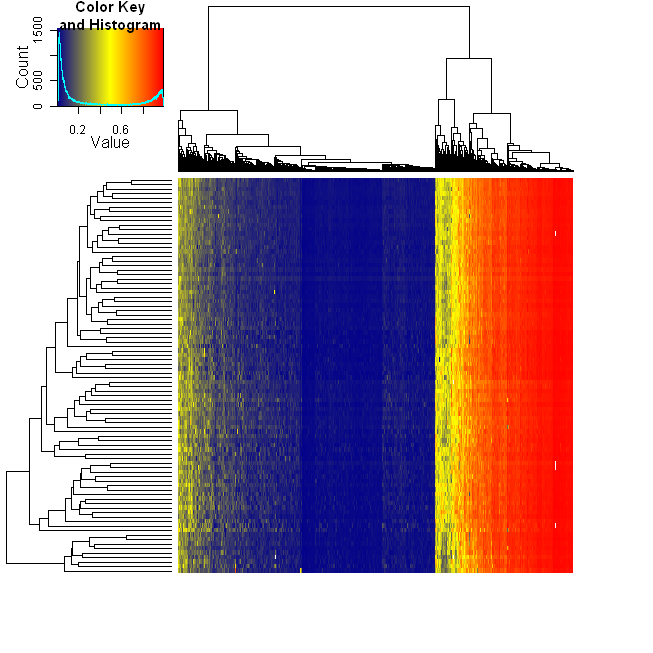

Supplement: Figure S1 — Heatmap of twin sample (1.28 MB TIF) [file pone.0006767.s006.tif]

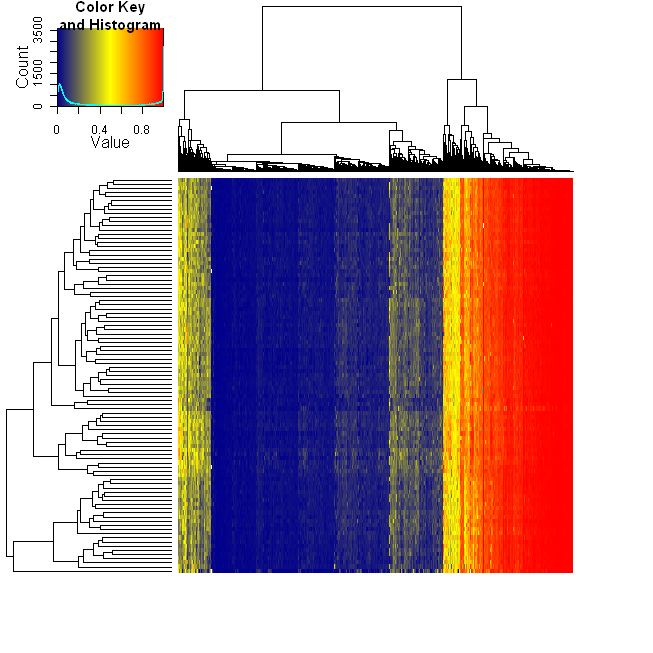

Supplement: Figure S2 — Heatmap of singleton sample (1.28 MB TIF) [file pone.0006767.s007.tif]
